# The Impact of Palliative Radiation Therapy on Patients With Advanced Gastric Cancer: Results of a Retrospective Cohort Study

**DOI:** 10.7759/cureus.32971

**Published:** 2022-12-26

**Authors:** Atsuto Katano, Hideomi Yamashita

**Affiliations:** 1 Radiology, The University of Tokyo Hospital, Tokyo, JPN

**Keywords:** external beam radiation, advanced cancer, palliative radiation therapy, gastric malignancy, supportive and palliative care

## Abstract

Aim

In advanced gastric cancer, symptoms such as loss of appetite, stomach tightness, occasional pain, vomiting blood, and melena may occur. Palliative radiation therapy may be indicated in such cases. This study aimed to investigate the clinical outcomes of palliative radiotherapy in patients with advanced-stage gastric carcinoma.

Methods

From April 2018 to October 2022, consecutive patients with non-resected advanced gastric cancer who received radiation therapy for palliation of symptoms were included.

Results

A total of 23 patients with advanced-stage gastric carcinoma were analyzed in this study. Twelve male and 11 female patients were included. The median overall survival period was 3.9 months (95% confidence interval: 1.0-8.7 months). Sixteen patients required erythrocyte transfusion before radiotherapy; for 13 patients (83%), the required units of erythrocyte transfusion decreased after palliative radiotherapy. The mean erythrocyte transfusion units significantly decreased from 4.2 (standard deviation [SD]: 4.3) to 1.7 (SD: 3.6) (p = 0.02). No adverse events of grade ≥3 were observed in this study population.

Conclusion

Palliative radiation therapy for advanced gastric cancer yielded a good response rate and can be a useful treatment option.

## Introduction

Although the worldwide incidence of gastric cancer is declining, there are large regional differences in the incidence rates of gastric cancer, with East Asia, including Japan, having a particularly high incidence [1]. The major risk factors for gastric cancer include bacterial infection, smoking, diet, and alcohol consumption [2]. In recent years, next-generation sequencing technologies have elucidated the landscape of genetic variations in the carcinogenesis of gastric carcinoma [3].

Early-stage gastric cancer is often asymptomatic, but some patients may complain of mild gastric discomfort, heartburn, burping, loss of appetite, and nausea [4]. Patients with advanced gastric cancer experience several symptoms such as loss of appetite, stomach tightness, occasional pain, vomiting of blood, and melena [5].

Surgical resection of the bleeding lesion seems to be the most direct way to relieve symptoms [6,7]. However, it often requires combined resection of the surrounding tissues and is a highly invasive procedure. Cowling et al. reported that palliative resection is associated with a high complication rate [8]. Radiation therapy is one of the palliative local therapies indicated for advanced gastric cancer. In this study, we report the results of palliative radiotherapy in patients with gastric cancer at our hospital.

## Materials and methods

We reviewed consecutive patients with non-resected advanced gastric cancer who underwent palliative radiation therapy between April 2018 and October 2022 to relieve symptoms such as tumor bleeding or obstruction. All patients were diagnosed with pathologically proven gastric adenocarcinoma. The clinical stage was determined according to the Union for International Cancer Control Staging, 8th edition. The institutional review board of our hospital approved this research. All patients provided informed consent to participate in this study.

**Figure 1:**
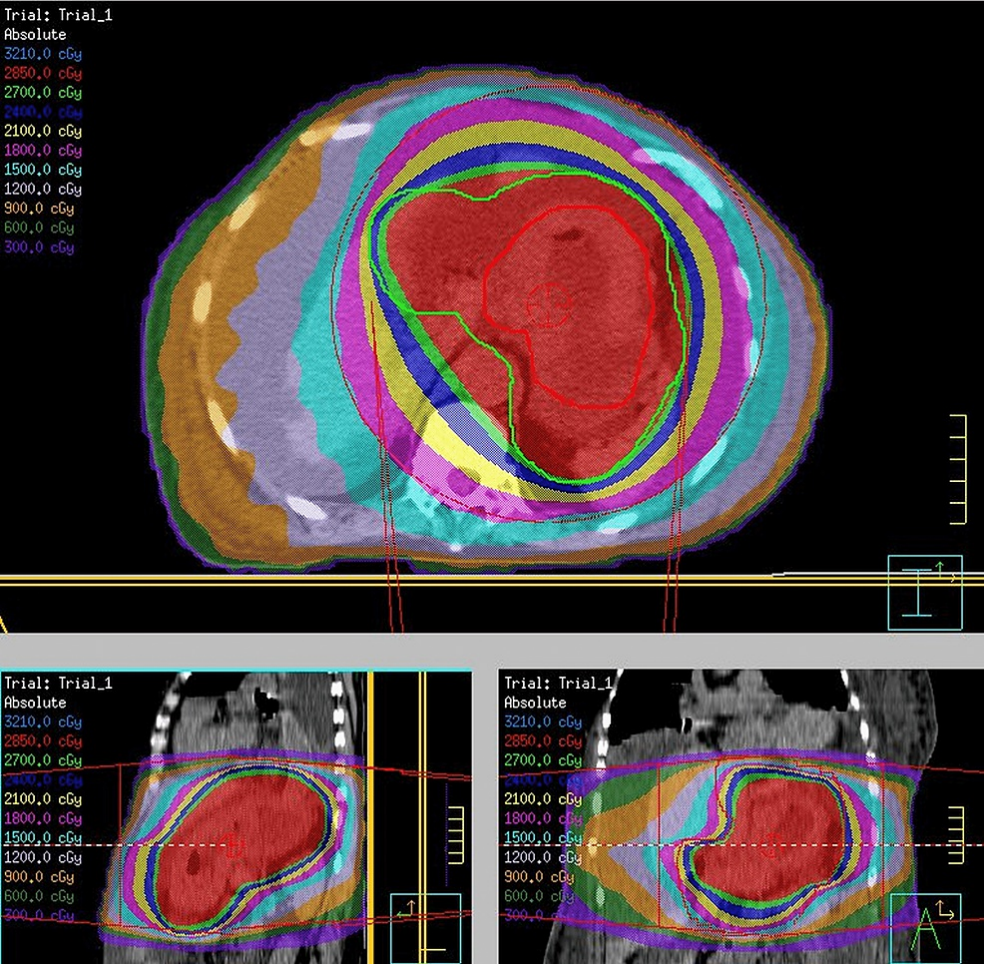
Radiation treatment planning The dose distribution of palliative radiation therapy of gastric cancer Isodose lines is described in the upper left corner.

All patients were treated with three-dimensional conformal radiation therapy (3D-CRT), without using intensity-modulated radiation therapy. The clinical target volume (CTV) was defined as the entire stomach on the planned CT scan. The internal target volume (ITV) was obtained by expanding the CTV based on the results of the respiratory motion assessment. The planning target volume (PTV) was defined as a uniform expansion of the margins by 5 mm around the ITV. The basic method was four-field or conformal arc radiotherapy with a total dose of 30 Gray (Gy) in 10 fractions, delivered with 10-megavolt x-rays (Figure 1), but the dose was adjusted according to the general condition and performance status.

The units of erythrocyte transfusions were recorded before and after palliative radiotherapy. After palliative radiotherapy, the units of erythrocyte transfusions were assessed for one month after the completion of palliative radiotherapy. Overall survival (OS) was calculated as the period from the date of initiation of radiotherapy until the date of death due to any cause or until the last date of follow-up. Survival curves were analyzed using the Kaplan-Meier method. R statistical software was used for statistical analysis. Statistical significance was set at p-value < 0.05.

## Results

Twenty-three patients were included in the study. The patient characteristics are presented in Table 1.

**Table 1:** Basic characteristics This table shows the characteristics of 23 patients who underwent palliative radiation therapy for advanced gastric cancer. ECOG-PS: Eastern Cooperative Oncology Group Performance Status Scale.

Variables	Number (Percentage)
Age: Median	77 (Range: 47-90)
Gender	
Male	12 (52%)
Female	11 (48%)
ECOG-PS	
0	2 (9%)
1	17 (74%)
2	4 (17%)
Clinical stage	
III	3 (13%)
IV	20 (87%)
Radiotherapy	
30 Gy in 10 fractions	12 (52%)
20 Gy in 5 fractions	10 (43%)
8 Gy in 1 fraction	1 (4%)
Pathology	
Moderately differentiated	4 (17%)
Poorly differentiated/signet ring cell	18 (78%)
Undefined	1 (4%)

The male-to-female ratio was 12:11, and the median age was 77 years (range: 49-90 years). The Eastern Cooperative Oncology Group Performance Status was 0 in two patients, 1 in 17 patients, and 2 in four patients. Eighteen patients were diagnosed with undifferentiated or signet cell carcinoma and four with moderately differentiated carcinoma; there was no information about differentiation in the medical records of one patient. The clinical stage was IV in 20 patients and III in three patients. The median prescription radiation dose was 30 Gy, and a median of 10 fractions of radiation was delivered. Three patients received concurrent chemotherapy: two received a SOX regimen consisting of oxaliplatin and S-1, which is an oral fluoropyrimidine, and one received a FOLFOX regimen consisting of a combination of leucovorin and fluorouracil with oxaliplatin. The remaining patients did not receive any concurrent chemotherapy. None of the patients had undergone surgical or endoscopic treatment during the peri-radiotherapy period.

**Figure 2:**
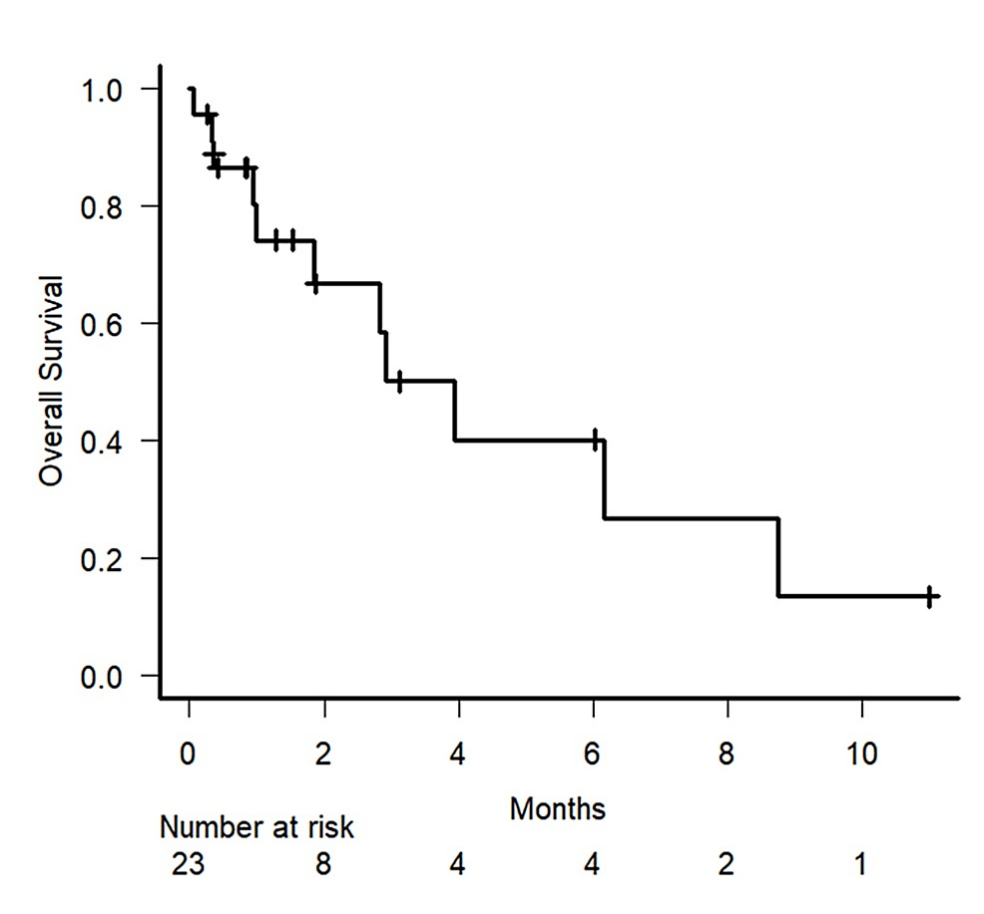
Kaplan–Meier plot Kaplan–Meier plot of the overall survival rate for the entire study cohort. The small vertical lines indicate a censored case.

The median OS in the present cohort was 3.9 months (95% confidence interval [CI]: 1.0-8.7 months). Figure 2 shows the Kaplan-Meier curves for the OS rate.

According to the results of univariate Cox regression analysis, no significant prognostic factor influenced the OS (Table 2).

**Table 2:** Univariate analyses for overall survival This table describes the univariate analyses of prognostic factors for overall survival using a proportional hazard model. Por: Poorly differentiated adenocarcinoma; Sig: Signet ring cell carcinoma.

Covariables	Comparison	Hazard ratio	95% CI	p-value
Age	>75 vs. <75 years old	0.299	0.087–1.032	0.056
Gender	Female vs. male	0.518	0.149–1.798	0.300
Performance status	2 vs. 1/0	3.218	0.714–14.500	0.128
Clinical stage	IV vs. III	2.829	0.351–22.800	0.329
Radiotherapy dose	20 Gy/8 Gy vs. 30 Gy	0.299	0.074–1.208	0.090
Pathology	Por/Sig vs. others	5.265	0.666–41.640	0.115

Symptoms from advanced gastric cancer were improved in 16 patients (70.0%). The mean serum hemoglobin concentration immediately before palliative radiotherapy was 9.0 g/dL (standard deviation [SD]: 1.8), and it slightly increased to 9.3 g/dL (SD: 1.7) after palliative radiotherapy; there was no statistically significant difference between both values (p = 0.27). In order to objectively evaluate the effectiveness of palliative radiotherapy for tumor bleeding, the amounts of erythrocyte transfusion required by the patient were compared before and after radiotherapy. Sixteen patients required erythrocyte transfusion before radiotherapy. Thirteen of the 16 patients (83%) required fewer units of erythrocyte transfusion during palliative radiotherapy than before. After palliative radiotherapy, the mean erythrocyte transfusion unit significantly decreased from 4.2 (SD: 4.3) to 1.7 (SD: 3.6) (p = 0.02). Adverse events included grade 1-2 nausea in nine patients and grade 1 esophagitis in one patient. No adverse events of grade ≥ 3 were recorded in this study.

## Discussion

We found that palliative radiotherapy was useful for the symptomatic palliation of advanced-stage gastric cancer. Palliative radiation therapy is well known to be effective in malignant bleeding in advanced cancer patients. However, there is no unified consensus on its specific efficacy, and further evidence needs to be accumulated to clarify the objective response rate. The effectiveness of palliative radiation therapy for gastric cancer has been recently reported as follows: Kawabata et al. treated 20 patients with unresectable gastric cancer with 3D-CRT [9]. There was a significant increase in the mean serum hemoglobin level (from 8.0 to 9.8, p = 0.01). Yu et al. administered radiation therapy to 61 patients with unresectable gastric cancer; out of them, 54 (88.5%) patients achieved bleeding control [10]. Takeda et al. demonstrated the hemostatic effect of palliative radiotherapy through a multi-institutional retrospective cohort study [11]. In a meta-analysis conducted by Tey et al., the pooled overall response rate for bleeding was 74% [12].

Transcatheter and endoscopic therapies are also useful palliative local therapy options for patients with advanced gastric cancer. Koh et al. reported a high rate of hemostasis using endoscopic therapy for bleeding from unresectable gastric cancer, especially from small lesions [13]. The technique they used included clipping, epinephrine injection/spray, and argon plasma coagulation. Recently, spraying of topical hemostatic agents by endoscopic application has been used for controlling gastrointestinal bleeding [14]. Cho et al. analyzed the data of 58 patients with gastric cancer bleeding at a single institution and found that the hemostatic rate when transcatheter arterial embolization was done was 72.4% [15].

Although combination chemotherapy with fluoropyrimidines and platinum-based agents has been used as systemic therapy for advanced or recurrent gastric cancer [16], some phase 3 trials have recently attempted to evaluate the efficacy of immune checkpoint blockade therapy as the first-line treatment. KEYNOTE-062 demonstrated the non-inferiority of single-agent pembrolizumab to conventional chemotherapy in patients with advanced-stage gastric cancer with a combined positive score (CPS) of 1 or greater with regard to OS (median OS: 10.6 versus 11.1 months) [17]. The ATTRACTION-4 trial compared chemotherapy plus placebo with chemotherapy plus nivolumab, and progression-free survival (PFS) had significantly improved in the nivolumab arm (median PFS: 8.3 versus 10.5 months, hazard ratio: 0.68; p = 0.0007) [18]. In the CheckMate 649 trial that included patients with unresectable gastric cancer of CPS 5 or higher, the median OS has significantly improved in the chemotherapy plus nivolumab group as compared to that in the chemotherapy group (median OS: 13.1 versus 11.1 months, hazard ratio: 0.71; p < 0.0001) [19]. Appropriate symptom palliation is more important than ever because longer survival can be achieved with these novel immunotherapies.

The size of the study cohort is the main limitation of the present study. There was no statistically significant difference in hemostatic effect or adverse events between the different radiation doses or fractions.

## Conclusions

In conclusion, we analyzed the use of palliative radiotherapy in patients with advanced gastric cancer at our hospital. Radiotherapy is a good treatment option for patients with advanced gastric cancer because it is less invasive than surgical or endoscopic approaches. Further accumulation of clinical data is needed to produce concrete evidence.
